# Effectiveness and safety of a one-yearly elongation approach of growing rods in the treatment of early-onset scoliosis: A case series of 40 patients with definitive fusion

**DOI:** 10.3389/fped.2022.895065

**Published:** 2022-11-18

**Authors:** Francesca Vittoria, Viola Ceconi, Lisa Fantina, Egidio Barbi, Marco Carbone

**Affiliations:** ^1^ Department of Surgery, Institute for Maternal and Child Health IRCCS Burlo Garofolo, Trieste, Italy; ^2^ University of Trieste, Trieste, Italy; ^3^ Department of Pediatrics, Institute for Maternal and Child Health IRCCS Burlo Garofolo, Trieste, Italy

**Keywords:** scoliosis, growing rods, early onset scoliosis (EOS), scoliosis correction surgery, distraction-based growth-friendly implants

## Abstract

**Background:**

Early onset scoliosis (EOS) can lead to marked spine and chest wall deformity and often to profound cardiopulmonary compromise. Nowadays, treatment benefits from the possibility of a growth-friendly surgical approach to avoid early spinal fusion. Growing rod spinal implants allow maximizing spine and thorax growth during childhood, performing lengthening procedures traditionally approximately every 6 months.

**Methods:**

We retrospectively evaluated 40 patients affected by EOS who underwent growing rod implantations from 2000 to 2020. A 1-year interval between lengthening procedures was adopted. Data about the age at the first and final surgeries, T1-T12 length pre- and post-surgery, T1-S1 pre- and post-surgery, major coronal curve, pre- and post-surgery rate of complications, and unplanned surgeries were collected and compared with those reported in the literature to determinate the effectiveness and safety of this long period between distractions.

**Results:**

The lengthening procedures were performed, on average, every 12.3 months; children underwent an average of 4.6 lengthening procedures each. Major curve pre-first surgery was 78°, post-first surgery 45°, pre-final surgery 55°, and post-final surgery 43°. The mean absolute difference between pre-initial to post-final major curve was 35°, representing a mean relative difference of 42%. On average, the T1-T12 segment measured 15 cm before the first surgery and 24 cm after the final surgery, while the T1-S1 segment was 25 cm before the growing rods implantation and reached 37 cm after treatment. During treatment, the adverse events affected 27 of the 40 total patients (67%) who experienced at least one complication. No differences were shown concerning both outcomes and complications, comparing these data with the available literature concerning most frequent elongations. This approach avoided four to five surgical procedures in this population.

**Conclusion:**

Our results related to deformity correction and complication rate are comparable with those found in the literature, where lengthening procedures are performed approximately every 6 months rather than with a 1-year interval between distractions. We also demonstrate a higher risk of complications for patients with implants before the age of 6.

## Introduction

*Early onset scoliosis (EOS)* is defined as a curvature of the spine ≥10° in the frontal plane with onset at 9 years of age or younger (1). EOS can be classified according to the patient’s age, major curve, eventually associated kyphosis, and etiology. As for the last variable, we can classify the curves into *idiopathic* without an evident causal agent; *congenital or structural*, which develop from a spine or thoracic cavity structural abnormality; *neuromuscular* with anomalies of muscle tone, which lead to scoliosis; and *syndromic* curves due to syndromes possibly associated with scoliosis, but not primarily related to structural or neuromuscular etiology (2). In the absence of treatment, ribs rotation and curve progression result in an increasing chest wall deformity. Lung function studies of children with EOS demonstrate a variable severity of restrictive lung disease caused by small lung volumes, reduced chest wall compliance, and respiratory muscle dysfunction (*thoracic insufficiency syndrome, TIS*) (3, 4). Therefore, untreated EOS is associated with significant morbidity and often profound cardiopulmonary compromise, including respiratory failure and cor pulmonale (5).

Current treatment strategies for EOS consist of nonoperative options and surgical treatments. Conservative strategies, *bracing* and *casting*, can be helpful, but often they represent only a temporary choice before surgery (6, 7). As far as surgery is concerned, great strides have been made over the past decade thanks to the so-called growth-friendly approach. There is relatively good evidence that EOS with early spinal fusion leads to pulmonary compromise and poor life quality (8, 9). Growth-friendly approach’s purpose is to allow and maximize the spine and thorax growth by controlling the curve’s progression. This method is achievable through three categories of spinal implants classified according to the correction forces exerted on the spine: *distraction-based*, *compression-based,* and *guided growth* (10).

Distraction-based implant systems are the most common devices used in EOS: a traction force is applied to the deformed spine segment with anchors at the top and bottom of the implants (on spine, ribs, or pelvis). Distraction-based implants are mainly *vertical expandable titanium rib prosthesis* (VEPTR), primarily made for children with TIS, and the most used growing rods, including both *traditional growing rods* and *magnetically controlled growing rods* (MCGR). MCGR are certainly promising, enhanced by the possibility of lengthening through noninvasive applied magnetic force without requiring general anesthesia, but they are not yet all employed and limited by the impossibility of performing MRI after implantation (1, 6, 10, 11). However, distraction-based techniques are burdened with a significant rate of complications, such as hook and screw dislodgement, rod fracture, prominent implant, wound infection, junctional kyphosis, and neurological complications (7, 12–15).

**Figure 1:**
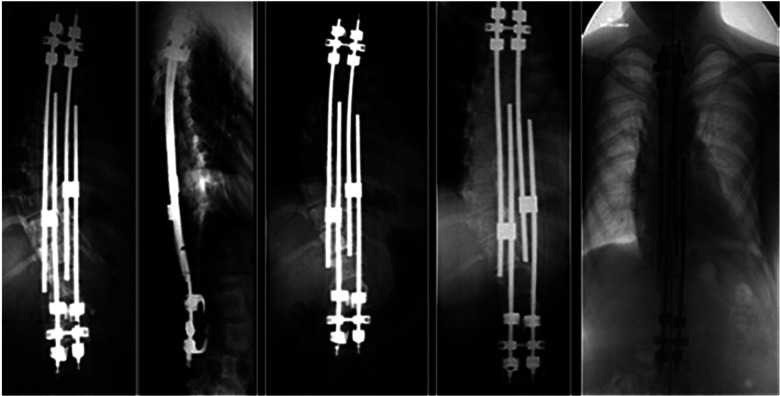
Four-year lengthened growing rods in a patient affected by Marfan’s syndrome.

Lengthening is traditionally carried out at 6-month intervals until the end of growth and after final definitive fusion surgery (7). The choice to intervene with lengthening at longer intervals can lead to savings in invasive surgical procedures, costs, risk of complication, and life quality. We reported our experience of distractions performed once a year in a cohort of patients affected by EOS and treated with growing rods techniques to evaluate this type of approach’s effectiveness and safety (Figure 1).

## Materials and methods

### Study design

For this retrospective study, data have been collected from 2000 to 2020 by the same medical team in two different institutes, “Institute for Maternal and Child Health” (Trieste, Italy) and “IRCCS Giannina Gaslini” (Genova, Italy). The study was approved by the Institute for Maternal and Child Health IRB (RC 34/18). Due to the retrospective nature of the study, informed consent signed by parents at the onset of the disease was adopted, in which they agreed that “the clinical data could be used for clinical research, epidemiology, disease study, and training purposes, to improve knowledge, treatment, and prevention.” In addition, all parents were requested to give specific, informed consent for the collection of the data.

### Objective of the study

The objective of the study is to analyze outcomes using the growing rod technique with a 1-year interval between distractions as surgical treatment of choice in patients affected by EOS, comparing results obtained with data available from the literature.

### Cohort of patients

**Figure 2:**
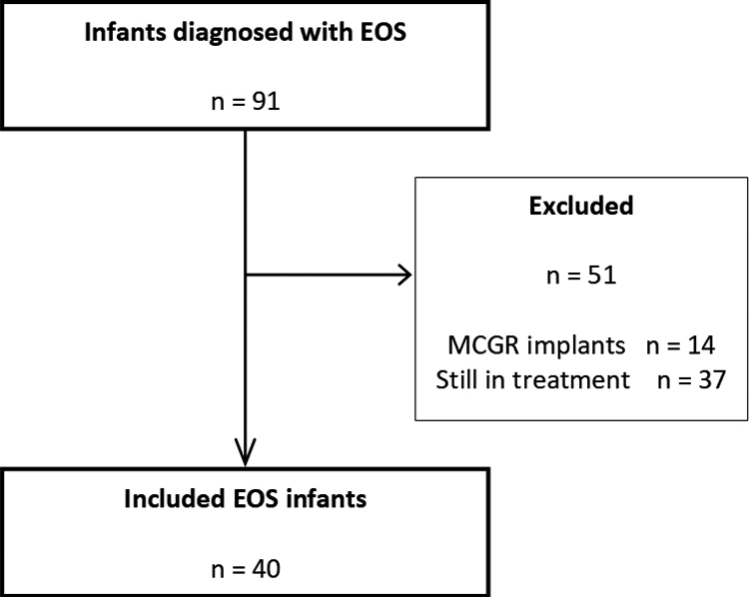
Flowchart of selected infants with EOS diagnosis treated with growing rods implant systems. EOS, early onset scoliosis.

The study cohort's inclusion criteria were patients affected by EOS who completed treatment with traditional growing rods with final spinal fusion surgery, regardless of their deformities’ etiology. In the 20 years of this study, 91 patients with EOS started surgical treatment by growing rod implantations. In this population, 14 patients received MCGR implant systems while 77 patients received the traditional dual growing rods. Of these 77 subjects, 40 completed their surgical treatment with the final spinal fusion and were included in the study cohort (Figure 2). Since, in the meantime, 37 patients of the initial 77 treated with traditional growing rods have not completed the surgical treatment with the spinal fusion and so were excluded, the final 40 patients who had been included in the cohort were not consecutive.

### Primary and secondary outcomes

The absolute improvement in T1-S1 segment length at the end of treatment was adopted as the primary outcome. Secondary outcomes were represented by deformity correction through major curve trends and safety estimates such as the rate of complications and the number of unplanned surgeries.

### Data collection and statistical analysis

We collected data on the age at first and final surgeries, interval time between distractions, eventually associated kyphosis, major curve pre- and post-first surgery, major curve pre- and post-distraction, major curve pre- and post-final surgery, T1-T12 length pre-first surgery and post-final surgery, T1-S1 pre-first surgery and post-final surgery, surgery duration, lengthening procedures, period of hospitalizations, complications, and unplanned surgeries. All these data have been analyzed on R 3.6.1, using descriptive statistics to calculate mean, standard deviation, median, and quartiles. Chi-squared tests and proportion tests were performed for examining relationships between variables, considering a *p*-value statistically significant <0.05.

## Results

### Patient cohort and demographic data

The examined cohort included 40 patients, 23 females and 17 males. Their deformities’ etiology was 14 syndromic, 10 congenital, 9 idiopathic, and 7 neuromuscular EOS. The mean age of the first surgery was 7.1 years, ranging from 2 to 11.8 years. Three patients had previously undergone an anterior and posterior hemiepiphysiodesis, and an anterior arthrodesis was performed on a child simultaneously to the first implantation surgery. One patient underwent Halo gravity traction before starting surgical treatment for 19 days. Twenty-five children presented kyphosis at the diagnosis.

The lengthening procedures were performed, on average, every 12.3 months, ranging from 9 to 23.5 months; children underwent approximately 4.6 lengthening procedures each, ranging from 0 to 11 procedures. Thirty-two patients finished their treatment with the growing rods, while 8 patients did not complete their therapeutic path. Three subjects changed the surgical approach for VEPTR implantation, and five underwent earlier definitive spinal fusion due to evolutive kyphosis complications (three were affected by congenital EOS, one by neuromuscular EOS, one by syndromic EOS).

The mean age at final spinal fusion is 14 years, ranging from 4.1 to 18.3 years. At the moment of the final surgery, 11 thoracoplasty procedures were necessary, with an average of five ribs. The requirement of osteotomies during the last surgery is not rare; indeed, 15 patients needed at least one. Two patients died after the end of treatment.

### Time for surgery, lengthening, and hospitalization

The average times required for the first surgery and last surgery was 238 min (range 135–390) and 434 min (range 105–590), respectively, while the average duration of a lengthening procedure was 54 min (range 39–77). The analysis did not include the instrumentation change and the correction of any complications that occurred.

The calculated medium time of hospitalization for the first and last surgery procedures was 17 (range 8–39) and 19 days (range 8–65), respectively, while the average hospitalization for a distraction procedure was 4 days (range 2–9) for each surgery (Table 1).

**Table 1:** Results in our 40-patient cohort.

Age at surgery (years)	
First surgery	7 (2–11)
Final surgery	14 (4–18)
Time needed for surgery (min)	
First surgery	238 (135–390)
Last surgery	434 (105–590)
Lengthening procedure	54 (39–77)
Time of hospitalization (days)	
First surgery	17 (8–39)
Last surgery	19 (8–65)
Lengthening procedure	4 (2–9)
Curve angles trend (°)	
Major curve	
Pre-first surgery	78 (35–116)
Post-first surgery	45 (19–65)
Pre-last surgery	55 (19–65)
Post-last surgery	43 (15–69)
Minor curve	
Pre-first surgery	71 (45–109)
Post-first surgery	43 (19–65)
Pre-last surgery	50 (11–82)
Post-last surgery	32 (2–49)
Segment lengths (cm)	
T1-T12 length pre-first surgery	15 (9–20)
T1-T12 length post-final surgery	24 (19–28)
T1-S1 length pre-first surgery	25 (16–32)
T1-S1 length post-final surgery	37 (30–41)

### Major curve trend

The major curves were measured before starting the treatment, resulting in an average of 78° (range 35–116). After the first surgery, a significant correction was obtained: the mean post-initial major curve was 45° (range 19–65). Before the last surgery, the curve degree increased again, reaching 55° (range 19°–65°); this phenomenon occurred physiologically in the time interval between the lengthening procedures. The final result once again showed an improvement in the angle 43° (range 15°–69°).

Fourteen patients had a minor curve; their pre-initial average major curve measured 71° (range 45°–109°). The correction achieved during the treatment was similar to the previous data. On average, the post-initial major curve measured 43° (range 19°–65°); in the pre-final angle, there was again an increase in the curve’s degree, reaching an average of 50° (range 11°–82°), which dropped significantly to 32° after the last surgery (range 2°–49°) (Table 1).

### Absolute and relative difference between pre-initial and post-final major curve

The mean absolute difference between the pre-initial and post-final major curve, calculated in 21 patients, was 35° (range 1°–74°), while the mean relative difference was 42% (range 4%–75%).

With regard to the minor curve, the mean absolute pre-initial and post-final difference measured 39° (range 5°–80°), corresponding to a mean relative difference of 53% (range 10%–96%).

There was a lack of data in the pre-initial and post-final major curve, which was not considered in calculating the absolute and relative mean differences, even though the expected results should have been similar.

### Segment lengths

On average, the T1-T12 segment measured 15 cm before the first surgery (range 9–20 cm) and 24 cm after the final one (range 15–28 cm). Eleven individuals presented both the pre-initial and post-final data about segment length, and the absolute improvement recorded in the T1-T12 segment was on average 7 cm (range 3–12 cm).

Our patients achieved similar results with the T1-S1 segment, starting with a mean segment of 25 cm before the GR implantation (range 16–32 cm) and gaining a mean 37 cm T1-S1 length after their treatment (range 30–41 cm). The mean absolute difference calculated between the post-final T1-S1 segment and the same pre-initial segment was 11 cm (range 5–19 cm) (Table 1).

### Complications

The adverse events met during treatment, related to the implantation of both traditional and magnetic growing rods, affected 27 of the 40 total patients (67%) who experienced at least one complication. Most children faced only one complication (48%), 30% two complications, 11% three complications, and 11% four or more complications. Rod breakage was the most frequent event, reported in 18 patients (eight patients with one rod breakage, six patients with two, two patients with three, one patient with four, and one patient with five). Six patients experienced anchors dislodgement, three patients wound dehiscence, three patients one or more decubitus, two patients bursitis, two patients cerebrospinal fluid leakage, one patient connector breakage, and one patient a dystrophy infection of the surgical site.

The percentage of subjects who suffered from at least one complication during treatment and underwent an unplanned surgery was 39%.

The differences found between children who underwent the first implant surgery before and after 6 years of age were also investigated. Twenty-five individuals formed the group of children who underwent first surgery after 6 years, and 14 of them reported at least one complication (56%), those below the age of 6 were 15 with an 87% rate of complications (*p*-value 0.0449).

## Discussion

One of the findings of this study is that children who underwent first implant surgery before 6 years of age had an increased risk of complications compared to older peers. In this perspective, different explanations could be considered. On the one hand, patients who needed to undergo surgical treatment so early in life may possibly have been more compromised and affected by rapidly evolving scoliosis. On the other hand, soft tissue coverage is more represented with advancing age, bones are larger, and physiological reserves enhanced. Moreover, it should be considered that the earlier in age children undergo initial rod implantation, the greater will be the number of elongation procedures required before the final spinal fusion. In 2010, Bess et al. demonstrated that for each surgical procedure performed in addition to the index surgery, there was an increased risk of complications. A patient who undergoing 7 procedures had a 49% chance of facing a complication; with 11 procedures, the complication risk increased to 80%. The complication rate increased by 24% for each additional procedure performed and decreased by 13% for each year of increased patient age at treatment initiation (12). Our results confirm that age older than 6 years at the time of initial rod implantation reduces the risk of adverse events during the treatment period.

In addition, this study demonstrated that once-a-year elongation allows similar results to twice-yearly elongation in EOS growing rods surgery and follow-up. Considering that complications were directly proportional to the number of distractions a child had to deal with and that the yield in spine growth from a given lengthening tends to decrease over time as a child undergoes more and more elongations (14), other studies recommend enlarged intervals between lengthening procedures. In a retrospective multicenter study, Paloski et al. analyzed two groups of patients based on how many months had passed between distractions: the first group with less than 9 months and the second with 9 months or more. Subjects with longer times between growing rod distractions had no significant differences in the primary Cobb angle, T1-S1 length, or instrumented length gain compared with patients with shorter times between distractions (16). Nevertheless, at this time worldwide, the most widely used approach for growing rod procedures provides for 6-month intervals between elongation procedures, consistent with most studies available in the literature (7, 10, 14, 17).

We are aware of the intrinsic limits of a direct comparison of data from different study populations. However, we found similar results in terms of effectiveness and safety with a 1-year lengthening procedure approach, except for a single study showing a significantly limited complication rate (11) (Table 2). Psychological consequences due to more frequent surgery procedures and hospitalizations are relevant too. Fear and anxiety are common issues in children undergoing repeated frequent surgical procedures (18, 19) and possible adverse effects on cognitive, academic, emotional, and sociobehavioral outcomes should be considered. In the given case of EOS, it was demonstrated that there was a higher rate of psychological dysfunction in these children (20), with a positive correlation between the number of repetitive surgeries and the behavioral problems (21).

**Table 2:** Comparison between our results and data available in the literature.

Study populations and outcomes	This study	Previous studies in the literature
Study populations (no. of patients)	40	Akbarnia et al. (2005)	23
		Akbarnia et al. (2008)	13
		Watanabe et al. (2013)	88
		Thompson et al. (2005)	28
Pre-initial to post-final surgery Cobb angle difference (%)	42	Akbarnia et al. (2005)	54
		Akbarnia et al. (2008)	64
		Watanabe et al. (2013)	42
		Thompson et al. (2005)	71
Pre-initial to post-final surgery T1-S1 segment length (cm)	11	Akbarnia et al. (2005)	11
		Akbarnia et al. (2008)	12
		Watanabe et al. (2013)	—
		Thompson et al. (2005)	12
Rate of complications (%)	67	Akbarnia et al. (2005)	48
		Akbarnia et al. (2008)	43
		Watanabe et al. (2013)	57
		Thompson et al. (2005)	29
Rate of unplanned surgeries in patients who experienced at least 1 complication (%)	39	Akbarnia et al. (2005)	36
		Akbarnia et al. (2008)	—
		Watanabe et al. (2013)	—
		Thompson et al. (2005)	—

Finally, the economic burden should also be considered. Halving the number of hospitalizations and surgical procedures operating once a year instead of every six months and calculating an average cost of 5,000€ for every lengthening-finalized admission, with an average of 4.6 lengthening procedures for a subject, we would obtain a final saving of 23,000€ for each patient.

## Conclusion

This study that is about a 20-year experience of two centers in growth-friendly surgery for EOS confirms the finding that children who underwent index surgery at an older age show a lower rate of adverse event during the treatment period.

Moreover, it shows that a once-a-year lengthening approach is comparable to a twice-a-year procedure, with similar deformity correction and complication rate results. This approach could decrease the number of surgeries and hospitalizations, improve these children’s life quality, and reduce the risk of psychobehavioral consequences due to repetitive surgeries.

In conclusion, we suggest that a lengthening procedure sparing approach should be considered both by delaying, when possible, the initial implantation of growing rods and by limiting to once a year the number of elongation procedures before the final fusion surgery.

More data are needed to confirm these findings.

## Data Availability

The raw data supporting the conclusions of this article will be made available by the authors, without undue reservation.
